# Knowledge graphs as pedagogical bridges for symbolic reasoning in hybrid AI systems: a perspective

**DOI:** 10.3389/frai.2026.1739692

**Published:** 2026-06-12

**Authors:** Carlota Delgado Vera, Andrea Sinche-Guzmán

**Affiliations:** Facultad de Ciencias Agrarias, Universidad Agraria del Ecuador, Guayaquil, Ecuador

**Keywords:** AI education, explainable AI (XAI), knowledge graphs, neuro-symbolic artificial intelligence, symbolic reasoning

## Abstract

Artificial intelligence (AI) has achieved extraordinary progress in recent years, yet this progress reveals a deep educational and epistemic imbalance. Neural architectures have mastered prediction but often obscure the grounds of their outputs. This Perspective argues that knowledge graphs (KGs) are more than a technical advance: they are an intellectual bridge between symbolic and neural paradigms, and a pedagogical opportunity to reform university-level AI curricula. The true frontier of explainable AI is educational, not only technological. By reintroducing symbolic reasoning into advanced AI curricula and professional training, we can prepare students who design, build, deploy, and evaluate AI systems to understand and justify system outputs. The focus is higher education for future developers, deployers, and auditors of AI systems, not general AI literacy for everyday users of AI tools. Through historical analysis, theoretical synthesis, and pedagogical reflection, we show that knowledge graphs are not only computational infrastructures but also catalysts for cognitive transformation in how we teach, learn, and conceptualize intelligence.

## Introduction: the educational blind spot of artificial intelligence

In the last decade, deep learning has become the dominant language of artificial intelligence (AI). The proliferation of high-level frameworks such as TensorFlow and PyTorch has democratized access to powerful models (Abadi et al., 2016; Paszke et al., 2019), and pretrained model repositories and low-code interfaces have further reduced the need to implement models from first principles. However, this success has created a cognitive asymmetry. In many university computer science, data science, and engineering programs, students learn how to train models that predict with high precision but cannot readily explain their logic. The symbolic tradition of AI, once central to the identity of the field, has been marginalized to the point of invisibility. Systems such as MYCIN, an expert system for infectious-disease diagnosis, and DENDRAL, a system for inferring molecular structures from mass spectrometry data, once embodied a philosophy of intelligence that prized explanation and accountability (Lindsay, 1980; Shortliffe, 2012). Today, many curricula privilege efficiency and performance metrics over reasoning and transparency. This neglect has consequences that reach far beyond pedagogy. The focus of this perspective article is university-level AI curricula for students who will develop, deploy, or audit AI systems, not a claim that every AI tool user must master formal symbolic methods. When the study and practice of AI become driven almost exclusively by statistical learning, the field risks losing some of its philosophical depth and its capacity for ethical self-regulation. This concern is consistent with literature showing that AI literacy in higher education includes not only technical understanding but also critical appraisal and ethical reasoning, and that these dimensions do not automatically develop together (Bećirović et al., 2025; Laupichler et al., 2022; Wiese et al., 2025). This narrowing of perspective risks producing a technical culture in university AI programs where interpretability is treated as a secondary concern rather than an intellectual responsibility. The crisis of explainability, in other words, is also a crisis of formation. We are training highly capable model builders who may still lack the conceptual tools to interrogate what their models mean.

## From symbolic certainty to neural opacity

The history of AI is a pendulum swinging between precision and adaptability. In its early decades, symbolic AI offered a rigorous, rule-based conception of knowledge in which facts were explicitly encoded and decisions were derived through inference (Lindsay, 1980; Shortliffe, 2012). MYCIN, an expert system for infectious-disease diagnosis, could trace the rules behind a recommendation, while DENDRAL, a system for inferring molecular structures from mass spectrometry data, relied on transparent rule sets to narrow candidate structures (Lindsay, 1980; Shortliffe, 2012). This approach made AI systems auditable. Yet symbolic AI struggled with ambiguity, uncertainty, and the combinatorial explosion that arose as rule bases scaled. The symbolic era was rational but brittle.

The connectionist revolution that began in the 1980s reversed this paradigm. By the 2010s, with the rise of deep learning (LeCun et al., 2015), neural architectures such as GPT, a generative pretrained transformer for text prediction (Radford et al., 2018), and BERT, a bidirectional encoder model for language understanding (Devlin et al., 2019), achieved striking generalization across domains. Their success demonstrated that useful capabilities could emerge from data rather than be imposed entirely by hand-coded design. Yet these models introduced a new opacity. Their internal representations were statistically coherent but often semantically opaque, and they could hallucinate facts or reproduce biases. They predict with confidence but cannot directly expose the reasoning path behind a given output. As educators of university students who will work with AI systems, we cannot treat this as a purely technical issue. The eclipse of symbolic reasoning in curricula means that new generations of practitioners may come to think of intelligence only as correlation, not comprehension.

This imbalance, we believe, has shaped the epistemology of AI in higher education. The shift from logic to learning has transformed both how intelligence is conceptualized and how it is taught. Students are trained to optimize accuracy, but they are given fewer opportunities to examine representation, justification, and inference. Interpretability can therefore appear as an external constraint rather than an intrinsic property of responsible system design. Yet without the capacity to explain how a result was produced and under what assumptions it should be trusted, prediction loses part of its moral and cognitive legitimacy.

## The hybrid turn: knowledge graphs as cognitive scaffolds

The current movement toward hybrid or neuro-symbolic AI is not simply a technical correction; it represents a philosophical return to balance. Research now seeks to combine neural adaptability with symbolic precision (Bhuyan et al., 2024; Vidal et al., 2025). Liu et al. (2025) distinguish three modes of integration: embeddings that encode knowledge graphs (KGs) into vector spaces, neural models constrained by symbolic rules, and architectures that combine reasoning modules with learning networks. Each relies on KGs as connective tissue between meaning and data.

Hybrid architectures are also present in deployed educational systems where LLM outputs are paired with deterministic tools to enforce correctness constraints. For example, Khan Academy reports building a calculator for Khanmigo to solve numerical problems rather than relying on probabilistic number prediction in a language model, an approach used to improve accuracy in math tutoring (DiCerbo, 2025).

Knowledge graphs (KGs) encode facts as subject-predicate-object triplets, establishing semantic networks that machines and humans can interpret alike. They are foundational to modern AI systems such as Google Knowledge Graph, a semantic layer for search (Singhal, 2012), ConceptNet, a commonsense knowledge base (Speer et al., 2017), and Wikidata, a collaboratively edited structured knowledge repository (Vrandečić and Krötzsch, 2014). Their value lies in how they make relationships explicit. They transform data into meaning by structuring the world as a system of interdependent relations.

In our experience as educators of university students in AI-related courses, the visual and relational nature of KGs makes them useful for teaching abstract reasoning. When students see how entities connect through defined predicates, they grasp that knowledge is not a list of attributes but a system of relations. When a machine learning course incorporates a short module on knowledge representation using RDF, a standard framework for describing linked data, OWL, a language for formal ontologies, or SPARQL, a query language for graph-structured data (Cyganiak et al., 2014; Harris et al., 2013), students begin to appreciate that models are not only computational devices but also epistemic instruments. They learn that a well-constructed ontology can constrain a neural system to behave more coherently. At this intersection between logic and learning, university AI curricula can recover core philosophical questions about representation, justification, inference, and the conditions under which an AI output deserves trust.

## A pedagogical framework for symbolic thinking

Building on Yang et al. (2023), we propose a three-layer pedagogical framework that uses KGs to cultivate reasoning, reflection, and responsibility in university AI curricula. The first layer, representation, invites students to visualize knowledge. In this stage, KGs function as cognitive maps. Each node corresponds to a concept, and each edge describes a relationship. For example, a course graph could map how calculus connects to differential equations and ultimately to machine learning. Such visualizations make dependencies visible and invite students to question why certain concepts connect. When learners perceive the topology of knowledge, they begin to see learning itself as a structured process.

The second layer, reasoning, transforms these maps into dynamic thought processes. Students engage with the graph by tracing conceptual pathways, much as inference engines traverse linked data. When students explore how linear algebra supports neural networks, they practice symbolic reasoning through guided discovery. This mirrors diagnosis-oriented hybrid systems that use medical ontologies and knowledge graphs to justify predictions rather than merely output them (Gao et al., 2025). In teaching contexts, this practice fosters disciplined curiosity by training students to think relationally, not linearly.

The third layer, explanation, demands that learners reconstruct and justify their reasoning. Through SPARQL queries (Harris et al., 2013) or logic-based representations, they articulate why a particular inference holds. We have found that students who can express the logical foundation of an algorithm internalize a deeper sense of accountability. They no longer view explainability as a compliance task but as an intellectual virtue. This stage embodies the essence of explainable AI: not the automation of transparency but its cultivation as a cognitive habit. When a student can verbalize the reasoning behind a machine’s decision, they have crossed the threshold from using AI to understanding it. The emphasis on inference reconstruction aligns with work on deep comprehension, where understanding depends on integrating inferences into coherent mental models rather than relying on surface recall (Millis et al., 2018).

This triadic framework transforms university AI curricula into an exercise in epistemic awareness. It also provides a basis for assessment that distinguishes recall from strategic reasoning and extended thinking, consistent with Webb depth of knowledge framework (Webb, 1997). The goal is not merely to produce competent programmers but reflective practitioners who understand that AI outputs without justification remain intellectually and ethically incomplete. By positioning KGs as both learning tools and epistemic metaphors, we can teach students that the structure of knowledge itself is an ethical concern.

## Applied reflections: medicine, security, and education

The integration of knowledge graphs (KGs), graph-based representations of entities and their relations, into applied domains provides compelling analogies for education. In medicine, Gao et al. (2025) demonstrate that systems combining transformers with medical ontologies derived from UMLS improve diagnostic performance and interpretability. The same principle applies to students: reasoning supported by explicit knowledge structures enhances accuracy and accountability. In cybersecurity, Piplai et al. (2023) illustrate that threat detection systems using KGs not only identify anomalies but can justify why an action is suspicious. This mirrors the role of symbolic reasoning in education: every alert, every conclusion, must be explainable. In educational recommender systems, KGs personalize learning paths by linking concepts across domains, showing students why a given topic is relevant to their progress. The message is consistent across all contexts: understanding grows from structure.

Educational technology has a long lineage of hybrid systems that predate the current surge of large language models. AutoTutor, an intelligent tutoring system designed to hold natural-language dialogues with learners, illustrates how explicit symbolic dialogue control can be paired with statistical language processing while keeping evaluation criteria interpretable. AutoTutor implemented expectation-misconception tailored dialogue and used semantic pattern-matching methods such as latent semantic analysis and regular expressions to map open responses onto anticipated correct ideas and common misconceptions (Graesser, 2016). Work on conversation-based assessment similarly shows that rigor comparable to human interviewers is achievable when dialogue moves are designed to elicit targeted evidence, including demonstrations in language assessment contexts and more recent multi-agent architectures that integrate contemporary language models (Forsyth et al., 2019; Hou et al., 2025).

## The structural invisibility of symbolic knowledge

Despite their promise, symbolic methods remain comparatively invisible in many curricula. This invisibility is structural. It is easier to teach libraries than logics, to train models than to interpret them. High-level APIs, point-and-click interfaces, and automated pipelines can compress university AI curricula into workflow execution unless courses reconnect model use to underlying representations and inference.

Introducing symbolic reasoning requires institutional investment, teacher retraining, and curricular redesign. Many educators dismiss RDF or OWL as too abstract or outdated. Yet in doing so, we deny students access to the intellectual lineage that makes AI interpretable. We have seen classrooms where students can fine-tune GPT-based systems but cannot explain what an ontology is. This pedagogical gap perpetuates the technical opacity that policymakers now struggle to regulate.

The cultural inertia of the field reinforces this invisibility. In many research and deployment settings, interpretability remains secondary to performance. Industry incentives often favor speed over understanding. Industry incentives favor speed over understanding. Unless universities intervene, the next generation of AI developers will inherit not just powerful models but epistemically weak habits. A curriculum that prioritizes explainability would reframe success in AI not as predictive superiority but as cognitive clarity. Such a shift requires educators to see themselves not merely as technical instructors but as stewards of epistemic integrity.

## Curricular reform as the path to trustworthy AI

To change this trajectory, university AI curricula must be restructured around reasoning, not only modeling. Symbolic reasoning should occupy a foundational place in advanced AI programs. Courses in logic, ontological modeling, and graph-based reasoning must complement machine learning and data analysis. Assessment should reward the ability to explain decisions made by a model and critique its reasoning. Educators should be encouraged to use open knowledge graphs, such as ConceptNet (Speer et al., 2017) and Wikidata (Vrandečić and Krötzsch, 2014), to design assignments where students trace reasoning across nodes and relations.

Curricular reform is also a moral imperative. In a world where AI systems shape medical diagnoses, financial decisions, and judicial processes, transparency is not optional. Engineers must be taught that interpretability is not an aesthetic preference but an ethical duty. Global frameworks such as the EU’s AI Act and UNESCO’s AI ethics principles recognize this, but policy cannot substitute for pedagogy. The roots of trustworthy AI are in the classroom. Reforming curricula is not a question of adding new content but of reshaping intellectual priorities. We have come to see that every time we teach students to accept opaque models, we normalize opacity as a form of thought.

Reform also requires a new academic culture. University AI curricula should value reflection as much as technical competence. We must cultivate a generation of practitioners who view logic not as an obsolete artifact but as the grammar of accountability. The goal is not to return to the rigidity of early symbolic systems but to integrate the interpretive discipline of logic with the creativity of neural learning. This is the epistemic synthesis that the field has long sought, and education is where it begins.

## Conclusion

Knowledge graphs exemplify how technical design can become an educational philosophy. They teach us that intelligence is not merely the ability to predict but the capacity to explain. As frameworks that articulate relationships rather than obscure them, they remind us that meaning in AI is constructed, not merely computed. When incorporated into pedagogy, knowledge graphs allow students to perceive the structure of thought itself, to see how reasoning connects premises to conclusions and how understanding emerges from organized relations. In this sense, they serve both as a metaphor and as a method for cognitive transparency.

For advanced university AI curricula and professional training for developers and evaluators, reforming AI education through symbolic reasoning will not only produce more interpretable systems but also more reflective and ethically grounded practitioners. The true maturity of AI will not be measured only by the sophistication of its algorithms but also by the depth of understanding in those who design them. Trust alone is not sufficient: learners also need principled knowledge of how systems represent information, generate outputs, and fail. For that reason, the future of explainable AI begins in the classroom, where the cultivation of reasoning can become a foundation for more trustworthy intelligent systems.

## Data Availability

The original contributions presented in the study are included in the article/supplementary material, further inquiries can be directed to the corresponding author.
